# 非小细胞肺癌相关lncRNA的研究进展

**DOI:** 10.3779/j.issn.1009-3419.2018.01.06

**Published:** 2018-01-20

**Authors:** 亚琛 张, 迪 梁, 晶 靳, 聪敏 刘, 宇彤 贺

**Affiliations:** 050011 石家庄，河北医科大学第四医院肿瘤研究所 The Fourth Hospital of Heibei Medical University, Cancer Institute, Shijiazhuang 050011, China

**Keywords:** 长链非编码RNA, 肺肿瘤, 诊断, 治疗, 预后, LncRNA, Lung neoplasms, Diagnosis, Treatment, Prognosis

## Abstract

肺癌是世界上常见的恶性肿瘤之一，其发病率和死亡率都居全部恶性肿瘤的首位。长链非编码RNA（lncRNA）是一类转录长度超过200个核苷酸且无蛋白质编码功能的RNA，但在表观遗传学调控、细胞周期调控和细胞分化调控等众多生命活动中发挥重要作用。研究发现lncRNA在非小细胞肺癌（non-small cell lung cancer, NSCLC）组织和血液中异常表达，且对肿瘤细胞的增殖、迁移、侵袭和凋亡发挥了重要调节作用，与肿瘤的发生发展密切相关。探索lncRNA在NSCLC中的作用机制，有助于NSCLC的早期诊断，靶向治疗及改善预后。因此本文就lncRNA与NSCLC的发生、诊断、治疗和预后的最新研究进展做一阐述，以期为NSCLC的防治提供新思路。

## Abstract (en)

Lung cancer is one of the most important malignant tumors in the world. The morbidity and mortality rank the first in all kinds of cancer. Long non-coding RNA (lncRNA) is at least 200 nt long and has no protein coding capacity. It plays an important role in the epigenetic regulation, cell cycle regulation, the regulation of cell differentiation, and many other life activities. The studies indicate that dysregulation of lncRNAs in non-small cell lung cancer (NSCLC) tissue and blood circulation is associated with the occurrence and development of cancer. The lncRNAs play an significant role in proliferation, differentiation, migration and apoptosis of the tumor cells. Explore the potential mechanism between lncRNAs and NSCLC is beneficial for the early diagnosis, target therpy and improve prognosis. Therefore, the study aims to demonstrate the latest studies on the lncRNAs related to occurence, diagnosis, therpy and prognosis of NSCLC. It can help to deeply understanding of lncRNA, and provide new ideas for the prevention of NSCLC.

肺癌是世界上发病率和死亡率最高的恶性肿瘤，据世界卫生组织国际癌症研究中心（International Agency for Research on Cancer, IARC）资料^[1]^显示，2012年全世界约有182万肺癌新发病例，居全部恶性肿瘤发病的第1位，且我国肺癌的发病和死亡也非常严峻^[2-4]^。非小细胞肺癌（non-small cell lung cancer, NSCLC）是肺癌的主要类型，占所有肺癌的80%左右^[5]^。NSCLC主要分为鳞状细胞癌、腺癌、腺鳞癌、大细胞肺癌和肉瘤样癌等，由于早期缺乏明显临床表现，发现较晚，肿瘤细胞已发生淋巴转移和远处转移，因此预后较差，5年生存率通常低于20%^[6]^，严重威胁着人类的生命健康。因此研究NSCLC发生、发展的机制，对NSCLC诊断和治疗至关重要。随着分子生物学技术的发展，长链非编码RNA（long non-coding RNA, lncRNA）逐渐成为研究热点，为肿瘤的研究提供了新的方向。本文将对与NSCLC发生、诊断、治疗和预后相关的lncRNA研究进展做一阐述，以期为NSCLC的防治提供新思路。

## 1 lncRNA概述

LncRNA是非编码RNA的一个类型，由于转录长度超过200个核苷酸并且缺乏蛋白质编码能力而得名，在表观遗传学调控、细胞周期调控和细胞分化调控等众多生命活动中发挥重要作用^[7, ]^。研究^[9]^发现lncRNA在多种肿瘤中异常表达，且表达失调的lncRNA可作为肿瘤促进或抑制因子。它们具有编码蛋白质基因上游启动子区、干扰下游基因的表达、介导染色质重构及组蛋白修饰、与转录因子结合、与特定蛋白质结合及作为小分子RNA的前体分子等多种复杂的调控机制^[10, 11]^。大量关于NSCLC与lncRNA的研究显示，lncRNA可以影响多种信号通路，在NSCLC的形成和进展中起重要作用。

## 2 lncRNA与肺癌发病的关系

肺癌的发生与吸烟密切相关，特别是肺鳞癌的发生与吸烟极为密切，而我国是烟草消费大国，因吸烟导致的肺癌就占到了93.75%^[12]^。有研究者通过采用香烟烟雾提取物诱导人类支气管上皮细胞（human bronchial epithelial, HBE）发生癌变，研究结果发现，HBE中的结肠癌相关转录子1（colon cancer-associated transcript 1, CCAT1）和多梳组蛋白BMI1表达升高，且miR-218（肿瘤抑制因子）的表达水平降低。其潜在机制可能是，CCAT1降低了miR-218的表达水平，进而促进了BMI1的表达，影响了细胞周期和细胞转移能力^[13]^。该实验组进一步研究指出，致癌转录因子c-Myc可通过与CCAT1启动子区结合来激活CCAT1的表达，而CCAT1可通过绑定游离的miRNA let-7c促进c-Myc的表达，从而形成一个正反馈回路促进香烟烟雾提取物诱导细胞癌变过程^[14]^。

空气污染已成为我国严重的公共卫生问题，有研究表明，全球肺癌死亡病例中有12.8%归因与人类生产造成的空气污染^[15]^。宣威市是我国肺癌发病率较高的城市^[16]^，周光彪等^[17]^研究发现，在宣威市NSCLC患者肿瘤组织中有多种lncRNAs异常表达，其中lncRNA CAR intergenic 10（CAR10）的高表达尤为显著。进一步研究发现，煤炭燃烧产生的多环芳烃家族二苯并蒽可通过转录因子FoxF2促进CAR10在肺癌上皮细胞中的表达，高表达的CAR10与YB-1结合调节EGFR通路，从而促进细胞增殖、诱导肿瘤发生。在我国，与细颗粒物（PM_2.5_）污染相关的死亡人数占城市居民死亡的32%，死亡率为1.9‰^[18]^。Deng等^[19]^的研究结果表明，PM_2.5_暴露会引起细胞中活性氧（reactive oxygen species, ROS）和自噬能力的升高，表达升高的ROS可激活lncRNA loc146880的表达，并进一步上调细胞的自噬能力，从而增强了肿瘤细胞的迁移、侵袭能力。

职业暴露同样是NSCLC发生的重要危险因素，Zhou等^[20]^首次发现了母系表达基因3（maternally expressed gene 3, MEG3）与镍暴露导致的肺鳞癌有关。先前研究^[21-24]^表明，MEG3是一种肿瘤抑制因子，可通过多种途径抑制细胞增殖。该研究发现，镍暴露引起MEG3表达下调，其潜在机制可能是，镍暴露导致MEG3启动子区超甲基化和表达抑制，导致Akt/p70S6K/S6通路激活，从而引起人支气管上皮细胞的癌变。由此可见，lncRNA在环境有害物质暴露诱发的NSCLC发生中扮演着重要角色，对这些因素和通路的研究将有助于研究NSCLC发生的原因，为NSCLC的防治提供线索。

## 3 lncRNA的临床应用

### 3.1 与NSCLC诊断相关的lncRNA

虽然肺癌诊断技术在不断提高，但有学者指出仍有40%的NSCLC患者被诊断为局部进展期或晚期的肺癌，这些患者通常不能进行手术切除，预后较差^[25]^，因此急需探索有效、便捷、经济的诊断方法。近年来，对lncRNA的研究发现，lncRNA在NSCLC细胞中异常表达，与NSCLC的发生发展密切相关，且lncRNA具有稳定性、特异性和易获得性，表明lncRNA可能成为潜在的NSCLC生物诊断标记物。研究这些lncRNA在NSCLC组织、血液中的表达，有助于研发特异性诊断标志物，提高NSCLC患者的检出率。

#### 3.1.1 lncRNA在组织中的研究

许多研究发现，肺癌组织中表达异常的lncRNA和肿瘤的发生发展密切相关。Su等^[26]^研究了基因lncRNA PRAL（P53调控相关性lncRNA）对肺癌的影响，研究发现PRAL在肺癌组织中的表达明显低于癌旁及正常组织，且P53的表达水平也显著降低。将PRAL转染到NCI-H929和A549细胞系后，会促进P53的转录，抑制肿瘤细胞的增殖。据报道位于3p25.3的基因linc00312表达与肿瘤大小呈负相关且与NSCLC发生发展密切相关，Zhu等^[28]^研究了linc00312在NSCLC中的作用^[27]^发现，linc00312在肿瘤组织中表达下调，且与体外实验研究结果一致。研究还发现HOXA5转录因子和linc00312的表达呈正相关，说明linc00312可能是通过激活HOXA5转录因子的表达在细胞增殖、组织生长中发挥重要作用。最近，尿路上皮癌相关基因1（urothelial carcinoma associated 1, UCA1）已被确认为一种致癌基因，认为表达失调的UCA1与肿瘤发生发展紧密相关。有研究^[29]^发现，UCA1在NSCLC组织中高表达，且沉默UCA1后会降低肿瘤细胞的增殖能力。因此，进一步研究NSCLC组织中这些异常表达的lncRNA，将有可能使其成为潜在的生物诊断标志物，为NSCLC的诊断提供新的指标。

#### 3.1.2 lncRNA在血液中的研究

最近，循环长链非编码RNA成为肿瘤生物学标志物研究的焦点，研究认为其对癌症的诊断有非常重要的意义。Tang等^[30]^利用lncRNA芯片分析寻找血液中潜在的NSCLC生物标志物，发现了三种lncRNA（RP11-397D12.4, AC007403.1, ERICH1-AS1）表达均上调，且三种lncRNA的合并阳性预测值、阴性预测值分别为0.72和0.87，有望成为预测NSCLC发生的潜在生物标志物。同样，Hu等^[31]^研究发现三种循环lncRNA：SPRY4-IT1、ANRIL和NEAT1在肿瘤组织中的表达存在差异，ROC曲线分析其曲线下面积分别为0.603、0.798和0.693，此研究认为将三者结合到一起诊断效能将会大大提高，其灵敏度为82.8%，特异度为92.3%，曲线下面积（area under the curve, AUC）为0.876。可见，血液中异常表达的lncRNA可以进行NSCLC的诊断，且不同的lncRNA间具有相关性。此外，某些单一的lncRNA异常也有助于NSCLC的诊断，有研究发现lncRNA生长停滞特异性转录本5（growth arrest-specific transcript 5, GAS5）在肺癌组织中低表达，Liang等^[32]^研究发现在NSCLC患者的血浆中GAS5处于稳定状态且表达下调，其诊断灵敏度和特异度分别为82.2%和72.7%，AUC为0.909，确定了GAS5和NSCLC诊断的关系。Wang等^[33]^研究发现，UCA1在NSCLC患者的血浆中表达显著升高，且与肿瘤组织中的表达一致，AUC为0.886，因此，血浆UCA1同样可作为NSCLC诊断的潜在生物标志物，能提高NSCLC筛选效能。

### 3.2 与NSCLC治疗相关的lncRNA

lncRNA在NSCLC发生发展中扮演重要角色，相应的分子靶向治疗也在不断发展中。与其他靶向治疗机制类似，lncRNA的靶向治疗包括沉默、阻断、破坏致癌性的lncRNA，或向特定细胞中转入抑癌性的lncRNA。lncRNA具有多功能性，这一特点使它在疾病治疗方面拥有巨大的应用潜力，随着对lncRNA在肺癌发生发展过程中认识的加深，其在NSCLC治疗中的应用将越来越广泛。

#### 3.2.1 致癌lncRNAs

能促进细胞增殖、侵袭能力及转移过程，抑制细胞凋亡以促进肿瘤发生发展的lncRNA被称为致癌lncRNAs^[10]^。Yang^[34]^等发现lncRNA XLOC_008466在NSCLC患者中高表达。当抑制XLOC_008466表达后，会抑制细胞的增殖和侵袭能力，促进细胞凋亡。研究指出，XLOC_008466的功能与ceRNA类似，可直接结合miR-874使其表达下调，而MMP2和XIAP作为miR-874的下游靶点，表达将会升高，即XLOC_008466是通过miR-874-MMP2/XIAP通路来影响细胞的增殖和侵袭能力，从而发挥致癌作用的。因此，靶向药物通过阻断、破坏XLOC_008466可降低肿瘤细胞的增殖、侵袭能力，起到治疗的作用。还有研究发现与癌旁和正常组织相比，lncRNA Gm15290在NSCLC组织中明显高表达。将Gm15290转染到NSCLC A549细胞系中后，过表达的Gm15290可促进细胞的增殖和侵袭能力，当敲低Gm15290后，肿瘤细胞的增殖、侵袭能力明显减弱，且可以促进细胞凋亡。RNA pull-down试验证实了Gm15290可直接与miR-615-5p相结合，通过抑制miR-615-5p的表达并增加miR-615-5p靶基因（IGF2、AKT2和SHMT2）的蛋白质表达水平来促进肿瘤细胞增殖。因此，有学者使用miR-615-5p的类似物抑制了Gm15290对肿瘤细胞增殖、侵袭的作用，所以Gm15290将有望成为NSCLC治疗的关键靶点^[35]^。肺腺癌转移相关转录子1（metastasis associated lung adenocarcinoma transcript 1, MALAT1）可促进肿瘤细胞迁移、侵袭并肿瘤生长，在NSCLC发生发展中发挥重要作用。有学者利用小分子抑制剂组蛋白脱甲基酶JMJD1A与MALAT1基因启动子区相结合，抑制MALAT1的表达，从而抑制了肿瘤细胞迁移和侵袭能力^[36]^。据报道，抑癌基因失活在肿瘤发生发展中发挥重要作用，Zirong等^[37]^发现linc00473的高表达与抑癌基因LKB1在NSCLC中经常发生突变和失活有关。linc00473可作为LKB1失活导致的NSCLC的治疗靶点，为NSCLC的靶向治疗提供新的方向。

#### 3.2.2 肿瘤抑制lncRNAs

能抑制肿瘤发生发展的lncRNA被称为肿瘤抑制lncRNAs，发现新的肿瘤抑制lncRNAs并阐释他们的功能是了解肿瘤启动潜在机制的重要过程，且对进一步研发新的治疗靶点非常重要。反胰岛素生长因子2（insulin growth factor 2 antisense, IGF2AS）被认为是Wilms肿瘤中的印迹基因，参与多种蛋白质的转录和翻译^[38]^。研究发现IGF2AS在NSCLC组织中低表达，且和患者的总生存期密切相关。体外肺癌细胞系功能试验结果提示，高表达的IGF2AS明显抑制了肿瘤细胞的迁移能力。研究还探索了与IGF2AS相关的信号通路，发现IGF2AS表达上调会抑制IGF2/VEGF/bFGF信号通路，从而抑制NSCLC的发生发展，这表明IGF2AS是一个理想的药物治疗靶点^[39]^。HOTAIR是一种具有反式转录调控作用的lncRNA，可负向调节染色体转录，重组染色质并促进肿瘤进展。有研究发现，溴结构域抑制剂I-BET151，可通过影响HOTAIR启动子，降低HOTAIR的表达水平，从而起到肿瘤抑制的作用^[40]^。此外，在Kruer等^[41]^研究MEG3的作用机制中发现，通过使用帕博西尼治疗A549和SK-MES-1肺癌细胞后可活化Rb通路，增加MEG3的表达，而MEG3是通过影响Rb通路显著降低肿瘤细胞的增殖能力，为肺癌的治疗提供了一个潜在的方法。由此可见抑制性的lncRNA在NSCLC治疗方面同样具有广泛的应用前景，但还需要进一步研究探索从而更好的应用于临床。

#### 3.2.3 lncRNAs在放化疗中的作用

研究发现NSCLC患者中存在EGFR突变，这种突变对表皮生长因子受体酪氨酸激酶抑制剂（epidermal growth factor receptor tyrosine kinase inhibitors, EGFR-TKIs）如埃罗替尼和吉非替尼敏感^[42, 43]^，但是大约10%的患者在10个月-16个月内会出现耐药^[44]^。最近，有学者指出lncRNA在EGFR-TKIs耐药性中起重要作用。Ma等^[45]^的研究中发现，有多种lncRNA在EGFR-TKIs耐药的细胞中异常表达。其中在吉非替尼耐药的细胞系PC9中发现5种异常表达的lncRNA，包括3种高表达的lncRNA（UCA1, NEAT1, CASC9）和2种低表达的lncRNA（EWAST1, linc00524）。进一步研究lncRNA在EGFR-TKIs耐药性中的机制发现，CASC9和EWAST1共表达基因参与了几个重要途径，其中包括调节细胞生长、凋亡和染色质组装等途径，进而影响细胞对药物的敏感性。肺腺癌是NSCLC的主要类型，对化疗药物有较高的耐药性^[46]^。Pan等^[47]^研究多西他赛耐药的肺腺癌患者发现，linc-ROR在多西他赛耐药的细胞系中表达升高，体内实验表明，linc-ROR表达下调会提高患者对多西他赛治疗的敏感性，抑制肿瘤细胞的侵袭和扩散。进一步研究表明，linc-ROR可能是通过miR-145/FSCN1来影响EMT过程，从而影响肺腺癌患者对多西他赛的耐药性。放疗是肺癌治疗的重要手段之一，研究证实某些lncRNA和放疗敏感性相关。Xue等^[48]^研究发现在NSCLC组织和细胞中GAS5表达下调、miR-135b表达上调。试验结果显示，高表达的GAS5和低表达的miR-135b可显著降低照射下的NSCLC细胞的存活率，提高放疗敏感性，同时可通过抑制肿瘤细胞增殖、侵袭能力来显著抑制肿瘤的发生。Chen等^[16]^利用小鼠肺癌模型研究发现，经放射治疗后lncRNA HOTAIR表达下调，导致β-连环蛋白信号传导改变，最终抑制肿瘤细胞增殖。综上所述，CASC9、linc00277、linc-ROR、GAS5和HOTAIR等lncRNA均与NSCLC的治疗相关，同时可也作为NSCLC放化疗耐药性治疗的靶点，为NSCLC的治疗提供新思路。

### 3.3 与NSCLC预后相关的lncRNA

NSCLC患者由于早期缺乏特异的临床表现，发现时多已经处于晚期，甚至发生淋巴及远处转移，因此患者预后较差。如前文所述，多种lncRNA与肺癌的诊断和治疗相关，这有助于提高患者的预后。同时，相关的lncRNA也有望成为评估预后的重要因素。有研究发现SPRY4-IT1在NSCLC组织中表达明显降低，且SPRY4-IT1的表达水平和肿瘤大小（*P*=0.001）、病理分期（*P* < 0.001）、淋巴转移（*P*=0.003）密切相关，因此该研究指出SPRY4-IT1是NSCLC预后一个独立的危险因素（*P*=0.009）^[49]^。最新研究发现lncRNA NEAT1和MALAT1在NSCLC组织中高表达，并可以通过Oct4的调节促进肿瘤发生发展，且高表达的Oct4、NEAT1和MALAT1和NSCLC患者的不良预后有关，其HR=2.78（95%CI: 1.21-6.42）^[8]^。Tang等^[50]^探讨lncRNA在肺腺癌中的表达和预后的关系发现，有5种lncRNA（CYP4F26P、RP11-108M12.3、RP11-38M8.1、RP11-54H7.4和ZNF503-AS1）和肺腺癌的预后有关，其中CYP4F26P、RP11-108M12.3、RP11-38M8.1、RP11-54H7.4在肺癌组织中高表达，ZNF503-AS1低表达，这5种lncRNA预测患者5年生存期的AUC可到达0.691。多因素*Cox*回归分析指出HR为1.928（95%CI: 1.04-3.58），说明这五种lncRNA是肺腺癌预后一个重要指标。此外，lncRNA H19表达水平可在一定程度上反映肿瘤的侵袭性和转移状态^[51]^；还有研究发现，肿瘤手术治疗后lncRNA XIST和HIF1A-AS1血清水平显着降低，可作为评价治疗效果的标志物^[52]^。所以，对这些lncRNA的研究有利于评估NSCLC患者的预后，并为改善预后提供新的研究方向。

## 4 结语

近年来，肺癌的发病和死亡在不断增加，关于肺癌机制的研究也在不断进行中，随着深度测序、基因芯片、实时定量PCR检测和原位杂交等技术的快速发展和应用，相关lncRNA的研究也在不断深入，其在肺癌发生发展中的作用也备受关注。lncRNA在环境有害物质暴露诱发的NSCLC中发挥重要作用。据报道，CCAT1、CAR10、MEG3等lncRNA都会诱发支气管上皮细胞恶变，形成肿瘤。先前的研究已经证明多种lncRNAs在NSCLC组织和血液中异常表达，可以作为NSCLC诊断的标志物。不同于miRNA，lncRNA的表达水平能更好地反映疾病状态；同时，lncRNA的表达模式高度特异，提示其表达状态可以被用于疾病的诊断或分类。其中UCA1作为肿瘤发生发展的重要因子，在肿瘤组织和血液中的表达异常，使其有望成为有效的NSCLC诊断标志物，但在组织和血液中表达的稳定性和有效性还有待进一步研究。另外，不同种类的lncRNA也可作为NSCLC预后的标志物。通过研究lncRNA的异常表达，不仅可以评估患者肿瘤的转移情况，还可以评估患者治疗效果，从而综合评价患者预后。由此可见，研究这些lncRNA为NSCLC的诊断和预后提供了新的研究方向。

随着治疗手段的不断发展，靶向治疗成为癌症的有效治疗方法，lncRNA在靶向治疗中发挥重要的作用。如今，越来越多的lncRNA不断被发现，其中有致癌性lncRNA，如XLOC_008466、Gm15290、linc00473等，它们的过表达可促进肿瘤细胞的增殖、迁移和侵袭能力，促进肿瘤的进展，也有抑制性lncRNA，IGF2AS、MEG3、HOTAIR。此外，lncRNA的遗传多态性与化疗敏感性有关，也可作为肺癌患者预处理评估的生物标志物，改善化疗效果^[53]^。这些lncRNA不仅有望成为治疗药物的有效靶点，还在放化疗敏感性中发挥重要作用。研究这些lncRNA，了解他们在NSCLC发生发展中的机制，从而对NSCLC患者进行有效的治疗。随着抗肿瘤药物研发不断深入，对小分子化合物的研究已有所进展，为靶向治疗来了广阔的发展前景。但是，因为有关lncRNA的靶向治疗研究有限，尤其在临床应用方面，所以一方面需要进一步明确lncRNA的作用机制，探索更有效的靶点；另一方面需要探索lncRNA与肺癌传统治疗的关系及相互作用机制，为NSCLC的治疗提供新思路、新方法。

## References

[1] Ferlay J, Soerjomataram I, Dikshit R (2015). Cancer incidence and mortality worldwide: sources, methods and major patterns in GLOBOCAN 2012. Int J Cancer.

[2] Wang L, Yu C, Liu Y (2016). Lung Cancer Mortality Trends in China from 1988 to 2013: New Challenges, Opportunities for the Government. Int J Environ Res Public Health.

[3] Fitzmaurice C, Dicker D, Pain A (2015). The Global Burden of Cancer 2013. JAMA Oncol.

[4] Chen W, Zheng R, Zhang S (2017). Cancer incidence, mortality in China in 2013: an analysis based on urbanization level. Chin J Cancer Res.

[5] Siegel R, Naishadham D, Jemal A. (2013). Cancer statistics, 2013. CA Cancer J Clin.

[6] Allemani C, Weir HK, Carreira H (2015). Global surveillance of cancer survival 1995-2009: analysis of individual data for 25, 676, 887 patients from 279 population-based registries in 67 countries (CONCORD-2). Lancet.

[7] Chen JH, Zhou LY, Xu S (2017). Overexpression of lncRNA HOXA11-AS promotes cell epithelial-mesenchymal transition by repressing miR-200b in non-small cell lung cancer. Cancer Cell Int.

[8] Jen J, Tang YA, Lu YH (2017). Oct4 transcriptionally regulates the expression of long non-coding RNAs NEAT1 and MALAT1 to promote lung cancer progression. Mol Cancer.

[9] Zhang H, Chen Z, Wang X (2013). Long non-coding RNA: a new player in cancer. J Hematol Oncol.

[10] Ricciuti B, Mencaroni C, Paglialunga L (2016). Long noncoding RNAs: new insights into non-small cell lung cancer biology, diagnosis and therapy. Med Oncol.

[11] Kunz M, Wolf B, Schulze H (2016). Non-Coding RNAs in Lung Cancer: Contribution of Bioinformatics Analysis to the Development of Non-Invasive Diagnostic Tools. Genes.

[12] Wang JB, Jiang Y, Wei WQ (2010). Estimation of cancer incidence and mortality attributable to smoking in China. Cancer Causes Control.

[13] Lu L, Xu H, Luo F (2016). Epigenetic silencing of miR-218 by the lncRNA CCAT1, acting via BMI1, promotes an altered cell cycle transition in the malignant transformation of HBE cells induced by cigarette smoke extract. Toxicol Appl Pharmacol.

[14] Lu L, Qi H, Luo F (2017). Feedback circuitry via let-7c between lncRNA CCAT1, c-Myc is involved in cigarette smoke extract-induced malignant transformation of HBE cells. Oncotarget.

[15] Evans J, van Donkelaar A, Martin RV (2013). Estimates of global mortality attributable to particulate air pollution using satellite imagery. Environ Res.

[16] Lui KH, Dai WT, Chan CS (2017). Cancer risk from gaseous carbonyl compounds in indoor environment generated from household coal combustion in Xuanwei, China. Environ Sci Pollut Res Int.

[17] Wei MM, Zhou YC, Wen ZS (2016). Long non-coding RNA stabilizes the Y-box-binding protein 1, regulates the epidermal growth factor receptor to promote lung carcinogenesis. Oncotarget.

[18] Fang D, Wang Q, Li H (2016). Mortality effects assessment of ambient PM2.5 pollution in the 74 leading cities of China. Sci Total Environ.

[19] Deng X FN, Zheng M, Ye X. (2017). PM2.5 exposure-induced autophagy is mediated by lncRNA loc146880 which also promotes the migration, invasion of lung cancer cells. BBA-GEN SUBJECTS.

[20] Zhou C, Huang C, Wang J (2017). LncRNA MEG3 downregulation mediated by DNMT3b contributes to nickel malignant transformation of human bronchial epithelial cells via modulating PHLPP1 transcription, HIF-1alpha translation. Oncogene.

[21] Lu KH, Li W, Liu XH (2013). Long non-coding RNA MEG3 inhibits NSCLC cells proliferation and induces apoptosis by affecting p53 expression. BMC Cancer.

[22] Wang P, Ren Z, Sun P (2012). Overexpression of the long non-coding RNA MEG3 impairs in vitro glioma cell proliferation. J Cell Biochem.

[23] Zhang X, Gejman R, Mahta A (2010). Maternally expressed gene 3, an imprinted noncoding RNA gene, is associated with meningioma pathogenesis, progression. Cancer Res.

[24] Mondal T, Subhash S, Vaid R (2015). MEG3 long noncoding RNA regulates the TGF-beta pathway genes through formation of RNA-DNA triplex structures. Nat Commun.

[25] Ramalingam S, Belani C (2008). Systemic chemotherapy for advanced non-small cell lung cancer: recent advances and future directions. Oncologist.

[26] Su P, Wang F, Qi B (2017). P53 Regulation-association long non-coding RNA (LncRNA PRAL) inhibits cell proliferation by regulation of P53 in human lung cancer. Med Sci Monit.

[27] Yu H, Xu Q, Liu F (2015). Identification and validation of long noncoding RNA biomarkers in human non-small-cell lung carcinomas. J Thorac Oncol.

[28] Zhu Q, Lv T, Wu Y (2017). Long non-coding RNA 00312 regulated by HOXA5 inhibits tumour proliferation, promotes apoptosis in non-small cell lung cancer. J Cell Mol Med.

[29] Nie W, Ge HJ, Yang XQ (2016). LncRNA-UCA1 exerts oncogenic functions in non-small cell lung cancer by targeting miR-193a-3p. Cancer Lett.

[30] Tang Q, Ni Z, Cheng Z (2015). Three circulating long non-coding RNAs act as biomarkers for predicting NSCLC. Cell Physiol Biochem.

[31] Hu X, Bao J, Wang Z (2016). The plasma lncRNA acting as fingerprint in non-small-cell lung cancer. Tumour Biol.

[32] Liang W, Lv T, Shi X (2016). Circulating long noncoding RNA GAS5 is a novel biomarker for the diagnosis of nonsmall cell lung cancer. Medicine.

[33] Wang HM, Lu JH, Chen WY (2015). Upregulated lncRNA-UCA1 contributes to progression of lung cancer and is closely related to clinical diagnosis as a predictive biomarker in plasma. Int J Clin Exp Med.

[34] Yang R, Li P, Zhang G (2017). Long non-coding RNA XLOC_008466 functions as an oncogene in human non-small cell lung cancer by targeting miR-874. Cell Physiol Biochem.

[b35] 35Dong Y, Huo X, Sun R, et al. LncRNA Gm15290 promotes cell proliferation and invasion in non-small cell lung cancer through directly Interacting with and suppressing the tumor suppressor miR-615-5p. Oncol Res, 2017. doi: 10.3727/096504017X14930316817366

[36] Tee AE, Ling D, Nelson C (2014). The histone demethylase JMJD1A induces cell migration and invasion by up-regulating the expression of the long noncoding RNA MALAT1. Oncotarget.

[37] Chen Z, Li JL, Lin S (2016). cAMP/CREB-regulated LINC00473 marks LKB1-inactivated lung cancer, mediates tumor growth. J Clin Invest.

[38] Duart-Garcia C, Braunschweig MH (2013). The Igf2as transcript is exported into cytoplasm and associated with polysomes. Biochem Genet.

[39] Zhang X, Zhang X, Hu R (2017). Prognostic implication, functional role of long noncoding RNA IGF2AS in human non-small cell lung cancer. J Cell Biochem.

[40] Pastori C, Kapranov P, Penas C (2015). The Bromodomain protein BRD4 controls HOTAIR, a long noncoding RNA essential for glioblastoma proliferation. Proc Natl Acad Sci U S A.

[41] Kruer TL, Dougherty SM, Reynolds L (2016). Expression of the lncRNA maternally expressed gene 3 (MEG3) contributes to the control of lung cancer cell proliferation by the Rb pathway. PLoS One.

[42] Pan H, Jiang T, Cheng N (2016). Long non-coding RNA BC087858 induces non-T790M mutation acquired resistance to EGFR-TKIs by activating PI3K/AKT and MEK/ERK pathways and EMT in non-small-cell lung cancer. Oncotarget.

[43] Tan CS, Gilligan D, Pacey S (2015). Treatment approaches for EGFR-inhibitor-resistant patients with non-small-cell lung cancer. Lancet Oncol.

[44] Takeda M, Okamoto I, Fujita Y (2010). De novo resistance to epidermal growth factor receptor-tyrosine kinase inhibitors in EGFR mutation-positive patients with non-small cell lung cancer. J Thorac Oncol.

[45] Ma P, Zhang M, Nie F (2017). Transcriptome analysis of EGFR tyrosine kinase inhibitors resistance associated long noncoding RNA in non-small cell lung cancer. Biomed Pharmacother.

[46] Du L, Morgensztern D (2015). Chemotherapy for advanced-stage non-small cell lung cancer. Cancer J.

[47] Pan Y, Chen J, Tao L (2017). Long noncoding RNA ROR regulates chemoresistance in docetaxel-resistant lung adenocarcinoma cells via epithelial mesenchymal transition pathway. Oncotarget.

[48] Xue Y, Ni T, Jiang Y (2017). LncRNA GAS5 inhibits tumorigenesis, enhances radiosensitivity by suppressing miR-135b expression in non-small cell lung cancer. Oncol Res.

[49] Sun M, Liu XH, Lu KH (2014). EZH2-mediated epigenetic suppression of long noncoding RNA SPRY4-IT1 promotes NSCLC cell proliferation, metastasis by affecting the epithelial-mesenchymal transition. Cell Death Dis.

[50] Tang RX, Chen WJ, He RQ (2017). Identification of a RNA-Seq based prognostic signature with five lncRNAs for lung squamous cell carcinoma. Oncotarget.

[51] Zhou Y, Sheng B, Xia Q (2017). Association of long non-coding RNA H19 and microRNA-21 expression with the biological features and prognosis of non-small cell lung cancer. Cancer Gene Ther.

[52] Tantai J, Hu D, Yang Y (2015). Combined identification of long non-coding RNA XIST and HIF1A-AS1 in serum as an effective screening for non-small cell lung cancer. Int J Clin Exp Pathol.

[53] Gong WJ, Peng JB, Yin JY (2017). Association between well-characterized lung cancer lncRNA polymorphisms, platinum-based chemotherapy toxicity in Chinese patients with lung cancer. Acta Pharmacol Sin.

